# Risk of mortality in older adults with loss of appetite: An analysis of Medicare fee-for-service data

**DOI:** 10.1016/j.jnha.2023.100035

**Published:** 2024-02-02

**Authors:** Simon Dagenais, Sunday Clark, Roger A. Fielding, Cera Cantu, Sapna Prasad, Feng Dai, John D. Groarke

**Affiliations:** a Pfizer, New York, NY, USA; b Nutrition, Exercise Physiology and Sarcopenia Laboratory, Jean Mayer USDA Human Nutrition Research Center, Tufts University, Boston, MA, USA; c Clarify Health, San Francisco, CA, USA; d Pfizer, Cambridge, MA, USA

**Keywords:** Anorexia of aging, Loss of appetite, Claims, Medicare, Older adults

## Abstract

**Objectives:**

Prior research suggested that loss of appetite (LOA) among adults with Medicare fee-for-service (FFS) insurance in the United States increased the risk of mortality within 1 year; those findings were not adjusted for risk factors and confounders. The objective of this study was to compare the risk of mortality among Medicare FFS beneficiaries with LOA to a control group without LOA while controlling or adjusting for age, comorbidities, body mass index (BMI), and weight loss.

**Design:**

Retrospective and observational analysis of Medicare FFS health insurance claims data from October 1, 2015 to December 31, 2021.

**Setting:**

Claims from all settings (e.g., hospital inpatient/outpatient, office, assisted living facility, skilled nursing facility, hospice, rehabilitation facility, home) were included in these analyses.

**Participants:**

The LOA group included all individuals aged 65–115 years with continuous Medicare FFS medical coverage (Parts A and/or B) for at least 12 months before a claim with ICD-10 diagnosis code “R63.0 Anorexia”. The control group was drawn from individuals aged 65–115 years with continuous Medicare FFS coverage who did not have a diagnosis of R63.0. Individuals with LOA were matched 1:3 to those in the control group based on age, sex, and race/ethnicity.

**Measurements:**

Mortality in the LOA group was compared to mortality in the control group using Kaplan–Meier and Cox regression analyses and stratified or adjusted in terms of Charlson Comorbidity Index (CCI), claims-based frailty index (CFI), BMI, and weight loss.

**Results:**

The study population of 1,707,031 individuals with LOA and 5,121,093 controls without LOA was 61.7% female and 82.2% White. More individuals with LOA compared with the control group had a CCI score 5+ (52.4% vs. 19.4%), CFI score 5+ (31.6% vs. 6.4%), and BMI < 20 kg/m^2^ (11.2% vs. 2.1%). Median follow-up was 12 months (individuals with LOA) and 49 months (control group). In a matched population, the risk of mortality was significantly higher (unadjusted hazard ratio 4.40, 95% confidence interval 4.39–4.42) for individuals with LOA than the control group. Median survival time was 4 months (individuals with LOA) and 26 months (control group); differences in survival time remained when stratifying by CCI, BMI, and weight loss.

**Conclusion:**

Individuals with LOA had a substantially increased risk of death even after matching for age, sex, race/ethnicity, and adjusting for comorbidities. These findings highlight the burden of illness in older adults with LOA and the need for therapies.

## 1 Introduction

Loss of appetite (LOA) [1] is common in older adults—with prevalence estimates of 0.2%–55.0% in community-dwelling adults, 5.8%–61.0% for those in institutional care, and 13.0%–63.0% in inpatient settings [[2], [3], [4]]—but is often underrecognized or unmanaged by clinicians [5]. LOA has been associated with multiple adverse health outcomes including malnutrition, sarcopenia, poorer functional status, increased care needs, hospitalization, risk of falls, decreased health-related quality of life, and mortality [4]. Few studies have examined the epidemiology and burden of illness associated with LOA in the United States (US).

A previous study estimated that approximately 1% of US adults aged 65–115 years with Medicare fee-for-service (FFS) insurance were diagnosed with LOA (based on diagnosis codes reported in claims) each year [6]. Older adults with LOA had a higher comorbidity burden based on the Charlson Comorbidity Index (CCI) and an increased risk of mortality within 1 year compared with those without LOA [6]. However, those analyses did not examine the potentially confounding impact of comorbidities, baseline body mass index (BMI), and weight loss on the risk of mortality observed among those with or without LOA.

Given the high prevalence and prognostic relevance of LOA in older adults, there is increasing recognition of the need to screen for LOA in clinical practice and a call for better treatment options for those diagnosed with LOA [7]. Yet fundamental uncertainties persist about optimal patient selection and timing of interventions to address LOA. For example, what is the additional impact of LOA on mortality among older adults with multiple comorbidities? Is the impact of LOA on mortality different or more pronounced for older adults with a lower BMI while sparing those with a higher BMI? Should LOA be managed immediately upon diagnosis or only when weight loss becomes apparent?

The objective of this study was to examine the impact of LOA on mortality while accounting for risk factors or potential confounders in a large population of older adults in the US using real-world data (RWD).

## 2 Methods

### 2.1 Study design

This was an observational, retrospective study of administrative claims data among adults 65–115 years of age covered by the Medicare FFS health plan. Individuals with a diagnosis of LOA were matched to a control group of individuals without a diagnosis of LOA and compared in terms of comorbidities, frailty, and mortality. The overall study period was 75 months, from October 1, 2015, to December 31, 2021. Review by an ethics committee was not sought for this study because it relied on data that were commercially available, de-identified, and preexisting.

### 2.2 Data source

In the US, the federal health insurance program for adults aged 65 or older is termed Medicare and includes different components: Part A covers inpatient care, Part B covers outpatient care, and Part D covers prescription drugs. Individuals can choose to enroll in the Medicare program run by the US federal government, termed traditional or fee for service (FFS) Medicare, or enroll in a program run by private companies, termed Medicare Advantage or Part C Medicare. This study focused on claims data for Medicare FFS beneficiaries that were obtained from the Centers for Medicare and Medicaid Services (CMS) by Clarify Health (San Francisco, California) and analyzed through the Virtual Research Data Center (VRDC). The study database contained claims data for 100% of Medicare FFS beneficiaries, including medical, facility, professional, and prescription drug claims covered under Medicare FFS Part A, Part B, and Part D. The clinical information reported in claims included diagnoses based on International Classification of Disease, 10th edition (ICD-10) diagnosis codes, procedures based on ICD-10 procedure codes, Current Procedural Terminology procedure codes, and Healthcare Common Procedure Coding System procedure codes.

### 2.3 Study population

The study population included all Medicare FFS beneficiaries with an FFS claim between October 1, 2015, and September 30, 2021. Beneficiaries were followed through December 31, 2021, disenrollment from Medicare Part A and B, enrollment in Medicare Part C, or death, whichever event happened first.

LOA was identified using the ICD-10 diagnosis code “R63.0 Anorexia” in any position on at least one medical claim for any type of health encounter (e.g., evaluation and management, diagnostic testing, medical procedure) in any health care setting (e.g., outpatient, inpatient, skilled nursing facility). The ICD-10 diagnosis code R63.0 describes a clinical disorder characterized by anorexia, lack of appetite, LOA, or dysorexia, but excludes LOA from eating disorders such as anorexia nervosa (ICD-10 codes F50.00, F50.01, F50.02) and avoidant or restrictive food intake disorder (ICD-10 code F50.81), as well as LOA of nonorganic origin (ICD-10 code F50.89) [8].

The group with LOA were those individuals who had a claim with the ICD-10 diagnosis code R63.0 between October 1, 2016, and September 30, 2021, aged 65–115 years, continuous Medicare FFS medical coverage for at least 12 months before their first claim with the ICD-10 diagnosis code R63.0 (index date), no Part C enrollment for at least 12 months before their index date, and no claim with the ICD-10 diagnosis code R63.0 between October 1, 2015, and September 30, 2016.

The control group without LOA included those individuals who met eligibility criteria, had no claims with the ICD-10 diagnosis code R63.0 from October 1, 2015, to September 30, 2021, aged 65–115 years, had at least one Medicare FFS claim (index date) between October 1, 2016, and September 30, 2021, at least 12 months of continuous medical coverage before the index date, and no Part C enrollment in the 12 months prior to the index date.

Individuals with LOA were matched 1:3 with those in the control group based on age categories (65–69, 70–74, 75–79, 80–84, 85–89, 90–94, ≥95 years), sex (male, female), and race/ethnicity (Black, White, Hispanic, Asian, other).

### 2.4 Outcomes

The primary outcome of interest was probability of survival after the index date. A secondary outcome was the time interval between the index date and the date of death in the CMS Master Beneficiary Summary File. Outcomes were compared for individuals with LOA and the control group. The risk factors or potential confounders considered were comorbidities, frailty, BMI, and weight loss. The baseline period was defined as the 12-month period prior to an individual’s index date through 1 month post the index date. The year of incidence was the 12-month period after an individual’s index date.

Comorbidities were assessed using a modified CCI, with renal disease subdivided into mild/moderate or severe [9,10]. Frailty was assessed using a modified claims-based frailty index (CFI) [11] without the “anorexia” component. For both CCI and CFI, comorbidities were deemed present if at least one claim with a relevant diagnosis code or procedure code was identified during the baseline period; scores were categorized as none (0), mild (1–2), moderate (3–4), or severe (5+).

BMI during the year of incidence was based on having at least one claim with a relevant diagnosis code during the year of incidence. Categories of interest were BMI < 20 kg/m^2^ (ICD-10 R63.6, Z68.1), BMI 20.0–24.9 kg/m^2^ (ICD-10 Z68.20–Z68.24), BMI 25.0–29.9 kg/m^2^ (ICD-10 E66.3, Z68.25–Z68.29), and BMI ≥ 30 kg/m^2^ (Z68.30–Z68.44, E66.01, E66.09, E66.1, E66.2, E66.8, E66.9). Individuals with multiple claims related to BMI on the same date with conflicting BMI categories were excluded from the BMI analyses. Weight loss during the year of incidence was based on having at least one claim with a diagnosis code suggestive of weight loss (ICD-10 M62.50–M62.59 “Muscle wasting and atrophy”, M62.94 “Disorder of muscle, unspecified”, R63.4 “Abnormal weight loss”, R64 “Cachexia”) during the year of incidence.

### 2.5 Statistical analysis

Categorical data were presented descriptively with frequencies and proportions. Continuous data were presented with means and standard deviations. Differences in demographics and clinical characteristics between individuals with LOA and the control group were assessed using conditional logistic regression, using a discrete logistic model and forming a stratum for each matched set.

Survival analysis compared probability of survival and time from index date to date of death for individuals with LOA and the control group. Individuals were censored if they unenrolled from Medicare Parts A and B, enrolled in Medicare Part C, or were still alive at the end of the study period. Unadjusted Kaplan–Meier curves were used to visualize survival between individuals with LOA and the control group both overall and stratified by CCI category, BMI category, and weight loss. Log-rank tests were used to determine significant differences between LOA and control groups in the unadjusted Kaplan–Meier curves. Cox proportional hazard modeling was used to compare survival and estimate hazard ratios (HRs) and 95% confidence interval (CIs) as measures of effect size between individuals with LOA and the control group. To assess the contributions of risk factors or confounders that were unbalanced between individuals with LOA and the matched control group, a series of Cox regression models were conducted for census region, CCI category, CFI category, and key CCI comorbidities (malignancy, including leukemia and lymphoma; metastatic solid tumor; chronic pulmonary disease; congestive heart failure; mild or moderate renal disease; severe renal disease).

All statistical analyses were conducted in the VRDC using Statistical Analysis System (SAS) version 9.4 (SAS Institute, Cary, North Carolina); two-sided *p* < 0.05 was deemed statistically significant. Analyses and code were reviewed by a second analyst at Clarify Health for quality control purposes. Data were presented in accordance with relevant CMS guidelines.

## 3 Results

### 3.1 Study population

There was a total of 66,285,991 Medicare FFS beneficiaries with at least one medical claim between October 1, 2015, and September 30, 2021, including 1,707,601 with LOA and 34,986,466 without LOA who were eligible for matching in the control group (Fig. S1). When matching for age, sex, and race/ethnicity, 570 individuals with LOA (<0.1%) were excluded because no matched controls were available. The final study population included 1,707,031 individuals with LOA and 5,121,093 individuals in the control group (Fig. S1). Matching was successful in terms of age, sex, and race/ethnicity (Table S1). The mean (SD) age at index was 80.9 (8.7) years for individuals with LOA and 80.6 (8.8) years for the control group; groups were similar in terms of 5-year age bands (Table S1).

### 3.2 Baseline characteristics

A total of 668,823 (39.2%) individuals with LOA had at least one claim relevant to BMI, compared with 1,120,674 (21.9%) in the control group; a total of 4321 individuals were excluded from BMI analysis due to conflicting BMI claims on the same date. After matching, differences were noted for individuals with LOA and those in the control group in terms of census region, BMI category, weight loss, key CCI comorbidities, CCI category, and CFI category (all *p* < 0.001, Table 1). For example, a greater proportion of individuals with LOA compared with the control group, respectively, were from the South (44.7% vs. 38.1%), had BMI < 20 kg/m^2^ (11.2% vs. 2.1%), weight loss (40.3% vs. 6.7%), malignancy (35.4% vs. 19.8%), CCI score 5+ (52.4% vs. 19.4%), and CFI score 5+ (31.6% vs. 6.4%). Mean (SD) CCI score was 5.6 (4.0) for individuals with LOA and 2.6 (2.7) for the control group (Table 1).

**Table 1:** Demographic and clinical characteristics of population.

	Individuals with LOA (*n* = 1,707,031)		Control group (*n* = 5,121,093)		*p* Value^a^
	*n*	%	*n*	%	
**USA census region**					<0.001
Midwest	372,308	21.9	1,155,144	22.6	
Northeast	288,285	16.9	999,511	19.5	
South	761,976	44.7	1,951,675	38.1	
West	280,396	16.5	990,150	19.3	
Unknown	4,066	0.2	24,613	0.5	
**Bodymass index (kg/m^2^)**^b^					<0.001
<20.0	191,266	11.2	108,099	2.1	
20.0–24.9	199,532	11.7	226,435	4.4	
25.0–29.9	140,339	8.2	326,931	6.4	
≥30.0	135,686	7.9	459,209	9.0	
No BMI claim or multiple BMI claims on claim date	1,040,208	60.9	4,000,419	78.1	
**Weight loss**^b^					<0.001
No weight loss	1,019,202	59.7	4,777,926	93.3	
Weight loss	687,829	40.3	343,167	6.7	
**Key comorbidities**^c^					
Any malignancy, including leukemia and lymphoma	604,294	35.4	1,013,047	19.8	<0.001
Metastatic solid tumor	245,802	14.4	92,736	1.8	<0.001
Chronic pulmonary disease	672,949	39.4	1,127,216	22.0	<0.001
Congestive heart failure	589,021	34.5	875,294	17.1	<0.001
Mild or moderate renal disease	604,458	35.4	938,761	18.3	<0.001
Severe renal disease	142,411	8.3	143,884	2.8	<0.001
**CCI score**^c^					<0.001
Mean (SD)	5.6 (4.0)		2.6 (2.7)		<0.001
None (0)	92,962	5.4	1,342,422	26.2	
Mild (1–2)	344,374	20.2	1,740,038	34.0	
Moderate (3–4)	375,793	22.0	1,047,186	20.4	
Severe (5+)	893,902	52.4	991,447	19.4	
**CFI score**^c^					<0.001
None (0)	203,695	11.9	3,017,165	58.9	
Mild (1–2)	542,671	31.8	1,304,187	25.5	
Moderate (3–4)	421,350	24.7	474,213	9.3	
Severe (5+)	539,315	31.6	325,528	6.4	

Individuals with LOA (with a diagnosis of R63.0) were matched 1:3 to a control group (without a diagnosis of R63.0) based on age, sex, and race/ethnicity. Percentages may not total 100 due to rounding.

*Abbreviations*: BMI, body mass index; CCI, Charlson Comorbidity Index; CFI, modified claims-based frailty index; LOA, loss of appetite.

a *p* value from conditional logistic regression, using a discrete logistic model and forming a stratum for each matched set. The “no BMI claim” category, which included those individuals with multiple BMI claims on the claim date, was not included in the BMI analyses.

b During the year of incidence. The year of incidence was the 12-month period after an individual’s index date.

c Present at baseline. The baseline period was the 12-month period prior to an individual’s index date through 1 month post the index date.

### 3.3 Mortality

The median (range) duration of follow-up from index date to date of death or study censoring was 12 (0–62) months for individuals with LOA and 49 (0–62) months for the control group (Table S2). At the end of available follow-up, 55.3% of individuals with LOA had died, compared with 35.2% of the control group. Among those who died during the study period, the median (range) time from index date to date of death was 4 (0–62) months for individuals with LOA and 26 (0–62) months for the control group.

**Fig. 1:**
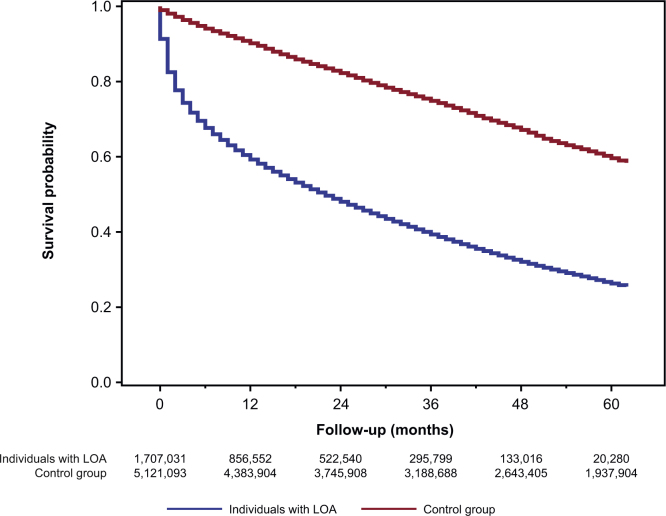
Survival of individuals with LOA and matched control group. *Abbreviation*: LOA, loss of appetite.

**Fig. 2:**
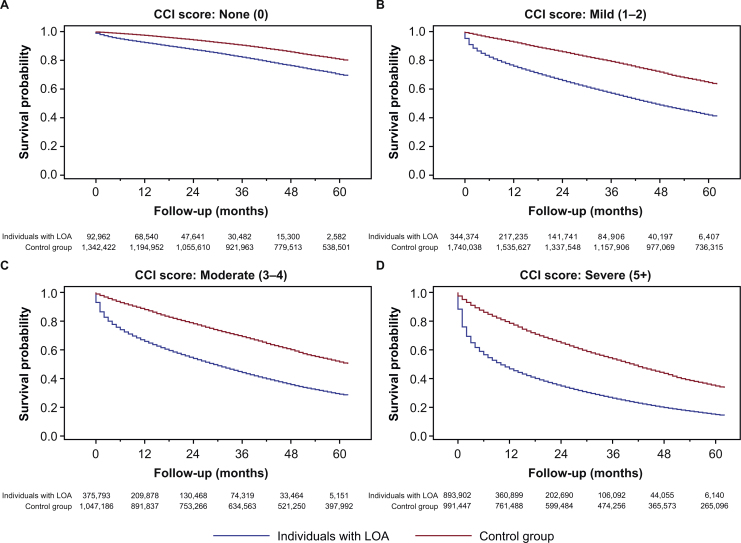
Survival of individuals with LOA and matched control group, stratified by CCI category at baseline: (A) CCI score 0, (B) CCI score 1–2, (C) CCI score 3–4, (D) CCI score 5+. *Abbreviations*: CCI, Charlson Comorbidity Index; LOA, loss of appetite.

**Fig. 3:**
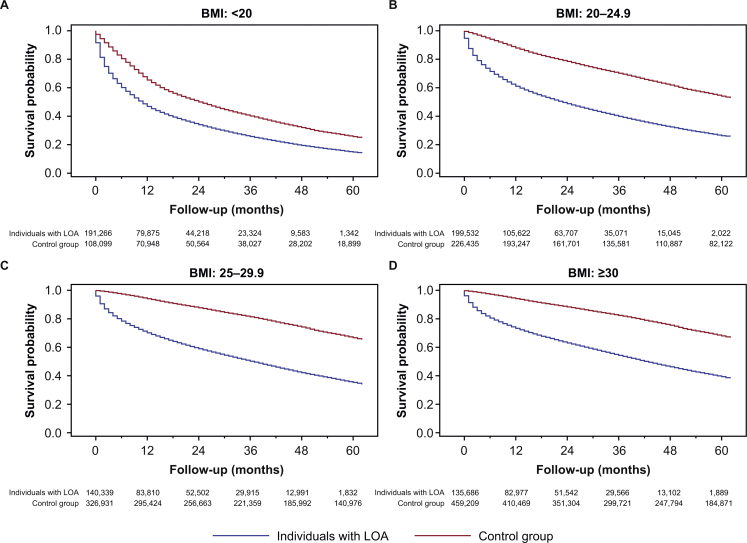
Survival of individuals with LOA and matched control group, stratified by BMI category during the year of incidence: (A) BMI <20 kg/m^2^, (B) BMI 20–24.9 kg/m^2^, (C) BMI 25–29.9 kg/m^2^, (D) BMI ≥30 kg/m^2^. *Abbreviations*: BMI, body mass index; LOA, loss of appetite.

**Fig. 4:**
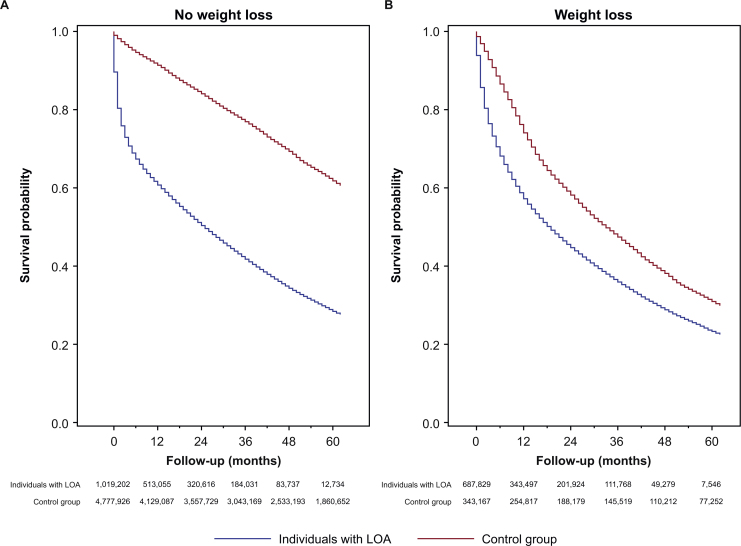
Survival of individuals with LOA and matched control group, stratified according to weight loss during the year of incidence: (A) without weight loss, (B) with weight loss. *Abbreviations*: CCI, Charlson Comorbidity Index; LOA, loss of appetite.

There were statistically significant differences (log-rank *p*-value < 0.001) in the probability of survival between individuals with LOA and the control group (Fig. 1). These differences in survival persisted between individuals with LOA and the control group even after stratifying by CCI category at baseline (Fig. 2), BMI category during the year of incidence (Fig. 3), or weight loss during the year of incidence (Fig. 4).

In a univariate Cox regression model, the risk of mortality was significantly increased in individuals with LOA compared with the control group, with a HR of 4.40 (95% CI: 4.39–4.42) (Table S3). The HR was broadly similar when controlling for differences in census region, malignancy, severe renal disease, or chronic pulmonary disease, suggesting these variables did not explain the differences observed in mortality. Slightly larger differences in the HR were noted in models that controlled for congestive heart failure or metastatic solid tumor, suggesting these variables did explain some of the difference in mortality. Of the variables considered, those with the greatest impact on HRs were CCI and CFI. The HR fell from 4.40 to 3.35 (95% CI: 3.33–3.36) when controlling for key comorbidities, to 3.20 (95% CI: 3.18–3.21) when controlling for CCI category, and to 2.66 (95% CI: 2.65–2.67) when controlling for CFI category, confirming that differences in comorbidities between groups explained some of the difference noted in mortality.

## 4 Discussion

In a large population of older adults in the US covered by Medicare FFS insurance during a 6-year period, individuals with a diagnosis of LOA had a risk of mortality that was more than fourfold higher than individuals in a matched control group without LOA. As expected, individuals with LOA were more likely to have a range of comorbidities included in the CCI or CFI indexes. Key CCI comorbidities that were believed to be associated with LOA and were therefore examined in these analyses included malignancies, metastatic solid tumors, chronic pulmonary disease, congestive heart failure, and renal disease. These findings are consistent with previous studies that reported adults with LOA had a higher burden of comorbidities and frailty [2,6,12,13]. Prior research found that LOA among adults with Medicare FFS insurance in the United States increased the overall risk of mortality within 1 year, but those findings were not adjusted for baseline risk factors and confounders [6]. The current analyses build on this prior research and were able to confirm that the increased risk of mortality with LOA remained even after adjusting, controlling, or stratifying by risk factors and confounders.

Although some of the difference observed in mortality for those with LOA versus those without LOA was explained by differences between groups in comorbidities after matching for age, sex, and race/ethnicity, the risk of mortality remained ∼3 times higher for individuals with LOA than the control group, even after controlling for comorbidities. Large differences in mortality remained even after stratifying by CCI category, BMI category, or weight loss category. These findings suggest that a diagnosis of LOA appears to be an independent risk factor for mortality among older adults in the US regardless of CCI or CFI comorbidities, BMI, or weight loss. These findings reinforce the importance of comorbidities on the health outcomes observed with LOA but suggest that comorbidities alone cannot fully explain the differences observed in mortality between the two study groups.

These findings are consistent with previous studies that reported LOA was an independent risk factor for mortality, which varied in their population and setting, as well as the method used to assess LOA [4,14]. While some clinicians may feel more comfortable relying on patient-reported questionnaires or clinical outcome assessments to identify LOA, analyzing the clinical information available in RWD allows researchers to examine much larger sample sizes [3]. Hence, our study population included nearly 2 million individuals with LOA. By examining clinical data that are not collected specifically for a clinical trial, RWD are likely more generalizable to the broader population of older adults in the US.

Previously, research based on Medicare FFS claims reported that individuals with a diagnosis of LOA based on the ICD-10 diagnosis code R63.0 had an increased risk of 12-month mortality (22.3%) compared with a control group without LOA (4.1%) [6]. Those analyses did not control for risk factors or potential confounders such as age, sex, race/ethnicity, comorbidities, BMI, or weight loss and were therefore unable to attribute the increased risk of mortality to the presence of LOA. The current analyses extend those earlier findings by adjusting, controlling, or stratifying by risk factors or potential confounders to demonstrate the independent association between LOA and mortality.

Although the BMI associated with increased longevity may be higher for older adults [[15], [16], [17]], our findings suggest that having a higher BMI may not protect older adults against the increased risk of mortality associated with LOA. The stratified survival analyses showed an association between LOA and mortality across all categories of BMI, including those with a BMI that could be classified as overweight (25–30 kg/m^2)^ or obese (≥30 kg/m^2^) [18]. This challenges a common clinical perception that LOA may be less clinically relevant in older adults with elevated BMI than in those with a low or normal BMI at baseline.

Unintentional weight loss in older adults has been associated with worse health outcomes [19]. Our stratified survival analyses showed a greater risk of mortality for individuals with LOA compared with controls, with or without weight loss. This suggests LOA may be prognostically important in older people regardless of subsequent weight loss. A previous study of community-dwelling older adults in Italy who were receiving home care reported the risk of mortality was higher among those with both anorexia and weight loss [20]. These observations suggest that clinical consideration to manage LOA in older adults should not be deferred until the onset of weight loss.

Although appetite and energy intake are often reduced in healthy older adults compared with younger adults [21], LOA should not be accepted as inevitable [5]. The pathophysiology of LOA in older people is the subject of ongoing research but is likely multifactorial, involving metabolic, physiologic, and social factors [1,2]. The inter-relationships between appetite and malnutrition, unintentional weight loss, sarcopenia, comorbidities, and frailty complicate research in this field. It is important to note that vitality – as measured by appetite and weight loss – is one of the five main body functions that contribute to an older adult’s intrinsic capacity for health, as outlined in various initiatives sponsored by the World Health Organization [7,[22], [23], [24]]. A better understanding of LOA will help in the quest to prevent and treat it, and studies of pharmacologic and non-pharmacologic interventions are needed [3,23]. The current data show that LOA should be considered as a therapeutic target irrespective of comorbidity burden, BMI, or weight loss.

There are some important limitations to acknowledge in this study. The retrospective and observational study design means that our findings can only report an association between LOA and mortality; causality cannot be established. The data are specific to the population of older adults covered by Medicare FFS in the US and may not be generalizable to those with other types of health insurance. Our working definition of LOA relied on the ICD-10 diagnosis code R63.0 being present on a claim. It is possible that this code is currently underutilized since it would likely not impact reimbursement, so individuals without such a claim could still have LOA. The ICD-10 diagnosis codes reported in claims could also represent working diagnoses that are later ruled out. Coding errors could also be present in a large administrative database that was not constructed specifically for research.

These limitations also apply to the identification of BMI and weight loss. Though we relied on ICD-10 diagnosis codes in claims to establish BMI, this information was only available in ∼40% of individuals with LOA and ∼20% of those in the control group without LOA. It is unclear if individuals with information about BMI available in claims data were representative of the overall population, and our analyses were unable to make this determination because we lacked more detailed clinical data. Nevertheless, our analyses of those with available data on BMI included ∼2 million individuals, which is a very large sample size. And while the ICD-10 diagnosis codes selected to indicate “weight loss” were suggestive of weight loss, our analyses were unable to confirm whether actual weight loss had occurred using longitudinal changes in bodyweight. Future studies with RWD should attempt to validate the clinical concepts developed from claims data by comparing them to other, more detailed sources of clinical information (e.g., data from electronic health records).

## 5 Conclusions

In this large retrospective study based on RWD in adults covered by Medicare FFS insurance, LOA was associated with a substantial increase in the risk of mortality even after adjusting for comorbidities. Large differences in mortality remained for individuals with LOA compared with matched controls when stratified by CCI, BMI, or weight loss. These data suggest that LOA should be considered clinically important in older adults irrespective of comorbidities, BMI, or weight loss. The role of therapeutics to manage LOA in older adults should be assessed in clinical trials.

## Author contributions

All authors contributed to the study design and conceptualization, data collection, data analysis, interpretation of data, and drafting of the manuscript.

## Funding information

The study was sponsored by 10.13039/100004319Pfizer. Medical writing support was provided by Kim Russell, PhD, of Engage Scientific Solutions (Horsham, UK) and was funded by Pfizer.

## Conflicts of interest

This study was supported by Pfizer Inc., New York, NY, USA. Simon Dagenais, Feng Dai, and John D. Groarke are employees of Pﬁzer with stock/stock options. At the time of the analyses, Sunday Clark was an employee of Pﬁzer with stock/stock options, and subsequently has Pfizer stock. Cera Cantu and Sapna Prasad are employees of Clarify Health Solutions, which received financial support from Pfizer for use of the Medicare claims database, consultation on study decisions, and data analysis. Outside of this work, Roger Fielding is partially supported by the US Department of Agriculture (USDA), under agreement No. 58-8050-9-004, by NIH Boston Claude D. Pepper Center (OAIC; 1P30AG031679). Roger A. Fielding has received travel support from Pfizer, consulting fees from Amazentis, Biophytis, GSK, Nestle, Pfizer, Regeneron, and Rejuvenate Bio, and owns stock or stock options in Axcella Health and Inside Tracker. Any opinions or recommendations expressed in this publication are those of the author(s) and do not necessarily reflect the views of the U.S. Department of Agriculture.

## Sponsor’s role

This study was funded by Pfizer (New York, NY). JG, SD, and FD are employees of Pfizer.

## Ethical standards

As this analysis only used commercially available de-identified data, formal review and approval by an institutional review board/independent ethics committee was not sought.
