# Supplementary material for: Risk of mortality in older adults with loss of appetite: An analysis of Medicare fee-for-service data
Source: J Nutr Health Aging. 2024 Feb 2;28(3):100035. doi: 10.1016/j.jnha.2023.100035 (PMC12880482; doi:10.1016/j.jnha.2023.100035)
Supplement: Supplementary file 1 [file mmc1.docx]

**SUPPLEMENTARY MATERIALS**

Table S1. Matching factors

Table S2. Follow-up and outcomes of individuals with LOA and matched control group

Table S3. Cox regression of mortality of individuals with LOA vs. matched control group

Figure S1. Flow diagram showing selection of the study population

Table S1. Matching factors

|  | **Individuals with LOA**  **(*n* = 1,707,031)** | | **Control group**  **(*n* = 5,121,093)** | |
| --- | --- | --- | --- | --- |
|  | ***n*** | **%** | ***n*** | **%** |
| **Age at index, years** |  |  |  |  |
| 65–69 | 193,943 | 11.4 | 581,829 | 11.4 |
| 70–74 | 278,216 | 16.3 | 834,648 | 16.3 |
| 75–79 | 303,238 | 17.8 | 909,714 | 17.8 |
| 80–84 | 315,084 | 18.5 | 945,252 | 18.5 |
| 85–89 | 296,939 | 17.4 | 890,817 | 17.4 |
| 90–94 | 214,898 | 12.6 | 644,694 | 12.6 |
| ≥95 | 104,713 | 6.1 | 314,139 | 6.1 |
| **Sex** |  |  |  |  |
| Male | 652,994 | 38.3 | 1,958,982 | 38.3 |
| Female | 1,054,037 | 61.7 | 3,162,111 | 61.7 |
| **Race/ethnicity** |  |  |  |  |
| Black | 186,154 | 10.9 | 558,462 | 10.9 |
| White | 1,402,428 | 82.2 | 4,207,284 | 82.2 |
| Hispanic | 35,607 | 2.1 | 106,821 | 2.1 |
| Asian | 46,584 | 2.7 | 139,752 | 2.7 |
| Other | 36,258 | 2.1 | 108,774 | 2.1 |

Individuals with LOA (with a diagnosis of R63.0) were matched 1:3 to a control group (without a diagnosis of R63.0) based on age, sex, and race/ethnicity. Percentages may not total 100 due to rounding.

Abbreviation: LOA, loss of appetite.

Table S2. Follow-up and outcomes of individuals with LOA and matched control group

|  | **Individuals with LOA**  **(*n* = 1,707,031)** | | **Control group**  **(*n* = 5,121,093)** | |
| --- | --- | --- | --- | --- |
|  | ***n*** | **%** | ***n*** | **%** |
| **Maximum follow-up (months)** |  |  |  |  |
| 0–5 | 593,935 | 34.8 | 424,749 | 8.3 |
| 6–11 | 256,544 | 15.0 | 312,440 | 6.1 |
| 12–17 | 192,234 | 11.3 | 361,023 | 7.0 |
| 18–23 | 141,778 | 8.3 | 276,973 | 5.4 |
| 24–29 | 123,916 | 7.3 | 310,481 | 6.1 |
| 30–35 | 102,825 | 6.0 | 246,739 | 4.8 |
| 36–41 | 87,380 | 5.1 | 291,923 | 5.7 |
| 42–47 | 75,403 | 4.4 | 253,360 | 4.9 |
| 48–53 | 60,920 | 3.6 | 325,933 | 6.4 |
| 54–62 | 72,096 | 4.2 | 2,317,472 | 45.3 |
| Mean (SD) | 17.3 (16.8) |  | 41.3 (21.6) |  |
| Median (minimum, maximum) | 12 (0, 62) |  | 49 (0, 62) |  |
| **Status at end of follow-up** |  |  |  |  |
| Loss of fee-for-service coverage | 509 | 0.0 | 4,351 | 0.1 |
| Began Medicare Advantage coverage | 85,703 | 5.0 | 636,776 | 12.4 |
| Alive | 676,772 | 39.6 | 2,679,673 | 52.3 |
| Dead^a^ | 944,047 | 55.3 | 1,800,293 | 35.2 |
| **Time from index date to death (months)** |  |  |  |  |
| Mean (SD) | 10.0 (12.5) |  | 27.3 (18.2) |  |
| Median (minimum, maximum) | 4 (0, 62) |  | 26 (0, 62) |  |

Individuals with LOA (with a diagnosis of R63.0) were matched 1:3 to a control group (without a diagnosis of R63.0) based on age, sex, and race/ethnicity. Percentages may not total 100 due to rounding.

Abbreviations: LOA, loss of appetite; SD, standard deviation.

^a^Does not include individuals that died after they lost fee-for-service coverage or enrolled in Medicare Advantage.

Table S3. Cox regression of mortality for individuals with LOA versus matched control group

|  | **Individuals with LOA vs.  control group** | |
| --- | --- | --- |
| **Model** | **HR** | **95% CI** |
| 1. Univariate model | 4.40 | 4.39–4.42 |
| 2. Controlling for census region | 4.39 | 4.38–4.41 |
| 3. Controlling for CCI categories | 3.20 | 3.18–3.21 |
| 4. Controlling for CFI categories | 2.66 | 2.65–2.67 |
| 5. Controlling for any malignancy, including leukemia and lymphoma | 4.39 | 4.38–4.41 |
| 6. Controlling for metastatic solid tumor | 3.98 | 3.96–3.99 |
| 7. Controlling for chronic pulmonary disease | 4.30 | 4.28–4.31 |
| 8. Controlling for congestive heart failure | 4.18 | 4.17–4.20 |
| 9. Controlling for mild or moderate renal disease | 4.29 | 4.27–4.30 |
| 10. Controlling for severe renal disease | 4.43 | 4.41–4.44 |
| 11. Controlling for CCI categories any malignancy, including leukemia and lymphoma; metastatic solid tumor; chronic pulmonary disease; congestive heart failure; mild or moderate renal disease; severe renal disease | 3.35 | 3.33–3.36 |

Individuals with LOA (with a diagnosis of R63.0) were matched 1:3 to a control group (without a diagnosis of R63.0) based on age, sex, and race/ethnicity. All Cox regression models included a variable to indicate individuals with LOA or control group. The non-univariate models added each covariate in independently.

Abbreviations: CCI, Charlson Comorbidity Index; CFI, modified claims-based frailty index; CI, confidence interval; HR, hazard ratio; LOA, loss of appetite.

Figure S1. Flow diagram showing selection of the study population

**
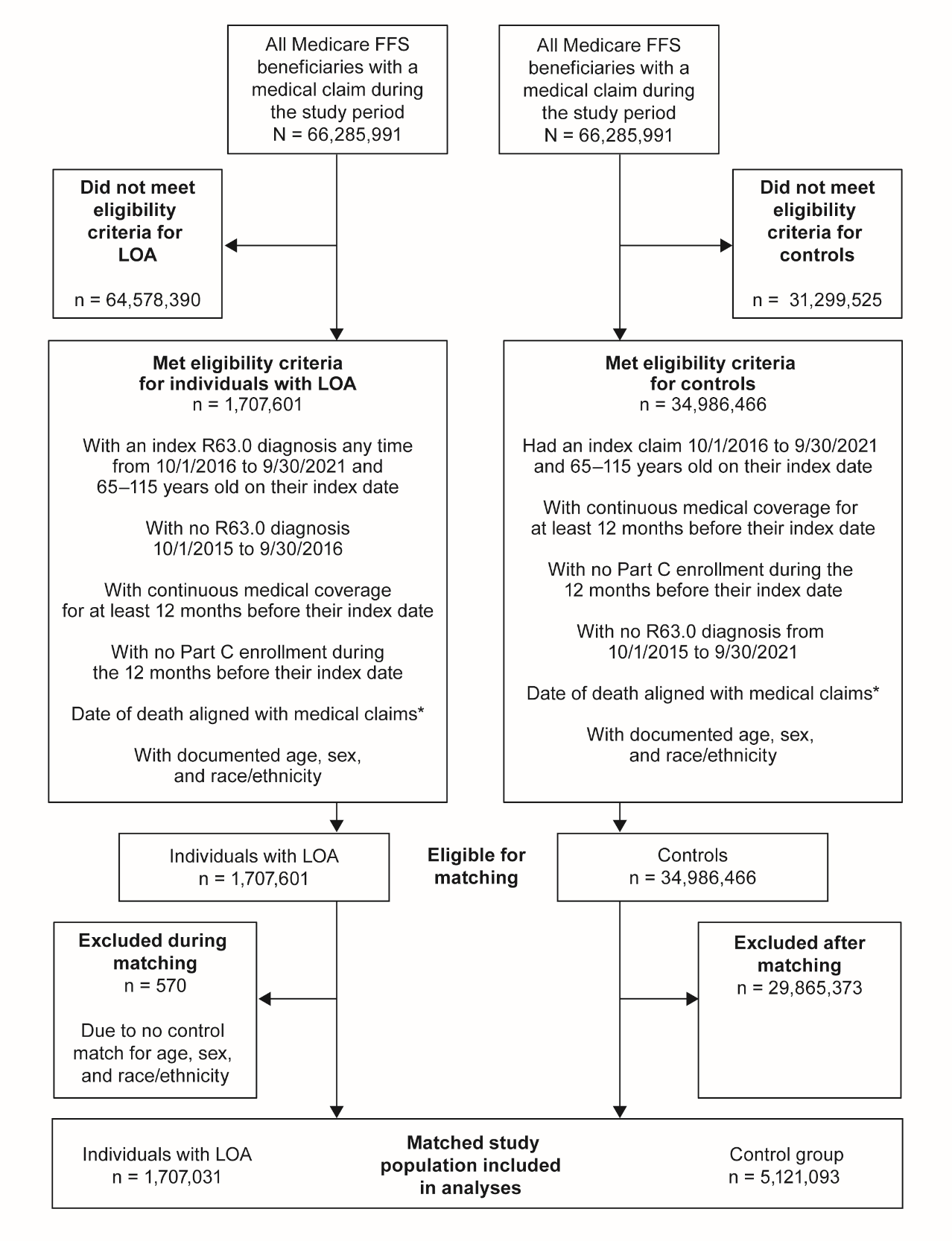
**

Individuals with LOA (with a diagnosis of R63.0) were matched 1:3 to a control group (without a diagnosis of R63.0) based on age, sex, and race/ethnicity. Dates are displayed in format Month/Day/Year.
*Where an individual had a documented date of death, this had to align with their medical claims.

Abbreviations: FFS, fee-for-service; LOA, loss of appetite.
